# Maternal oncostatin M levels in gestational diabetes mellitus: associations with metabolic dysfunction and pregnancy outcomes

**DOI:** 10.1590/1806-9282.20260975

**Published:** 2026-11-09

**Authors:** Bilge Kapudere, Yesim Akdemir, Berrak Guven

**Affiliations:** 1 Istanbul Medeniyet University, Department of Perinatology, Department of Obstetrics and Gynecology – Istanbul, Türkiye.; 2 Demiroglu Bilim University, Department of Obstetrics and Gynecology – Istanbul, Türkiye.; 3 Zonguldak Bulent Ecevit University, Department of Biochemistry – Zonguldak, Türkiye.

**Keywords:** Oncostatin M, Gestational diabetes mellitus, Insulin resistance, Pregnancy, Inflammation

## Abstract

**OBJECTIVE:**

Gestational diabetes mellitus is characterized by metabolic dysfunction and altered inflammatory signaling. The aim of this study was to evaluate maternal serum oncostatin M levels in pregnancies complicated by gestational diabetes mellitus and explore their associations with metabolic and pregnancy outcomes.

**METHODS:**

In this prospective case–control study, 40 women with gestational diabetes mellitus and 40 healthy pregnant controls were enrolled. Maternal serum oncostatin M concentrations were measured during the third trimester using an enzyme-linked immunosorbent assay.

**RESULTS:**

Maternal serum oncostatin M levels were significantly lower in women with gestational diabetes mellitus than in controls (28.7 vs. 64.0 ng/L, p=0.001). Oncostatin M levels were negatively correlated with body mass index, insulin, hemoglobin A1c, and Homeostatic Model Assessment of Insulin Resistance, and positively correlated with gestational age at delivery (all p<0.05). Pregnancy outcomes were similar between groups.

**CONCLUSION:**

Maternal oncostatin M levels are reduced in gestational diabetes mellitus and are associated with markers of metabolic dysfunction, supporting a potential association with the immunometabolic adaptations of diabetic pregnancy.

## INTRODUCTION

Gestational diabetes mellitus (GDM) is one of the most common medical complications of pregnancy and is associated with increased risks of adverse maternal and neonatal outcomes^1^. The disorder develops when pancreatic β-cell compensation becomes insufficient to overcome the progressive insulin resistance that normally occurs during pregnancy^2,3^. In addition to metabolic dysfunction, growing evidence suggests that chronic low-grade inflammation contributes substantially to the pathophysiology of GDM through its effects on insulin signaling, glucose metabolism, and placental function^1,4,5^.

Oncostatin M (OSM) is a pleiotropic cytokine belonging to the interleukin-6 family^6^. Beyond its established role in inflammatory regulation, OSM has been implicated in adipose tissue homeostasis, insulin resistance, and glucose metabolism^6–9^. Experimental studies have demonstrated that dysregulated OSM signaling may contribute to metabolic disorders, including obesity and type 2 diabetes, suggesting a potential role in the complex immunometabolic interactions underlying GDM^6,7^.

Pregnancy is characterized by profound endocrine, metabolic, and immunological adaptations^10,11^. OSM is expressed in decidual and placental tissues throughout gestation and has been implicated in placental endocrine activity^12^. Given the central role of placental adaptation and inflammatory signaling in the development of GDM, alterations in maternal circulating OSM levels may reflect disease-related changes in metabolic and placental function^1,12^.

Despite increasing interest in immunometabolic biomarkers during pregnancy, data regarding maternal OSM concentrations in GDM remain limited. Furthermore, the relationship between circulating OSM levels, metabolic parameters, and pregnancy outcomes has not been adequately investigated. Therefore, this study aimed to evaluate maternal serum OSM levels in pregnancies complicated by GDM and to explore their associations with metabolic characteristics and pregnancy outcomes.

## METHODS

This prospective observational study was conducted at the outpatient clinic of the Department of Obstetrics and Gynecology at Zonguldak Bulent Ecevit University Hospital. It included 40 pregnant women with GDM and 40 healthy pregnant controls. The institutional Ethics Committee approved the study protocol, and all procedures were performed in accordance with the principles of the Declaration of Helsinki. The study procedures were explained verbally to all participants, and written informed consent was obtained before participation. Due to missing data for some outcomes, the number of participants included in individual analyses varied slightly.

Routine screening for GDM was performed between 24 and 28 weeks of gestation using a 50-g oral glucose challenge test. Women with plasma glucose values above 140 mg/dL subsequently underwent a diagnostic 100-g oral glucose tolerance test using the Carpenter-Coustan criteria^4^. Demographic and clinical data, including maternal age, body mass index (BMI), and gestational age, were obtained from medical records. Women with multiple pregnancies or pre-existing chronic diseases were excluded. BMI was calculated as weight (kg) divided by height (m^2^).

Fasting venous blood samples were collected from all participants in the morning during the third trimester, without a predefined gestational week, using serum separator tubes. Serum was obtained by centrifugation at 3,000 *g* for 5 min and stored at −80°C until analysis. Serum OSM concentrations were measured using a commercially available enzyme-linked immunosorbent assay (ELISA) kit (Cloud-Clone Corp., Wuhan, China), in accordance with the manufacturer's instructions. Calibration was performed using serial dilutions of recombinant human OSM standards. The assay sensitivity was <6.3 pg/mL, and the intra-assay and inter-assay coefficients of variation were <10 and <12%, respectively.

Statistical analyses were performed using Statistical Package for the Social Sciences (SPSS) software for Windows (version 24.0; SPSS Inc., Chicago, IL, USA). Continuous variables were expressed as mean±standard deviation or median (minimum–maximum), as appropriate. Data distribution was assessed using the Kolmogorov-Smirnov test. Depending on normality, comparisons between groups were performed using Student's t-test or the Mann-Whitney U test. The Kruskal-Wallis test was used to compare more than two groups. Categorical variables were compared using the chi-square test or Fisher's exact test when appropriate. Associations between variables were evaluated using Spearman's rank correlation analysis. A p-value of <0.05 was considered statistically significant. Because no formal a priori sample size calculation was performed, an approximate post hoc power analysis was conducted based on the observed OSM differences between groups (n=40 per group), yielding 56.9% power (two-sided α=0.05; Cohen's d=0.48). This estimate should be interpreted cautiously because the primary comparison used the nonparametric Mann-Whitney U test. The primary multivariable logistic regression model included OSM, maternal age, BMI, and C-reactive protein (CRP). Sensitivity analyses were performed by individually adding glycated hemoglobin A1c (HbA1c), fasting insulin, Homeostatic Model Assessment of Insulin Resistance (HOMA-IR), ferritin, vitamin D, and low-density lipoprotein (LDL) to the primary model using complete cases available for each covariate.

## RESULTS

A total of 80 pregnant women were included in the study, comprising 40 women with GDM and 40 healthy pregnant controls. There were no significant differences between the groups in terms of maternal age (p=0.885), BMI (p=0.061), gravida (p=0.088), or parity (p=0.248). Smoking status was also comparable between the GDM and control groups (p=1.000).

Serum OSM levels were significantly lower in the GDM group than in controls (median 28.7 vs. 64.0 ng/L; p=0.001). Classical inflammatory markers, including CRP, alpha-1 antitrypsin, and ceruloplasmin, were similar between groups (all p>0.05). Similarly, HbA1c, HDL, and triglyceride levels did not differ significantly between groups. In contrast, OSM, neutrophil-to-lymphocyte ratio (NLR), and platelet-to-lymphocyte ratio (PLR) were significantly lower in the GDM group (p=0.001, 0.001, and 0.003, respectively). Among metabolic parameters, vitamin D, insulin, ferritin, and HOMA-IR were significantly higher in the GDM group (p=0.011, 0.019, 0.042, and 0.004, respectively). In contrast, LDL cholesterol was significantly lower (p=0.005), suggesting altered metabolic status in pregnancies complicated by GDM. The sample size for some laboratory parameters varied slightly due to missing data (Table 1).

**Table 1:** Comparison of serum oncostatin M, laboratory parameters, and perinatal and neonatal outcomes between the gestational diabetes mellitus and control groups.

		GDM (n=38)	Control (n=37)	p
OSM		28.7 (2.5–396.9)	64.0 (19.0–419.9)	**0.001** *
Inflammatory markers				
	PLR	106.88 (±45.6)	135.46 (±33.55)	**0.003** **
	NLR	3.16 (0.05–11.4)	4.22 (2.61–8.11)	**0.001** *
	CRP	7.10 (0.0–23.4)	5.97 (0.15–21.4)	0.478*
	Alpha-1 antitrypsin	224 (119–290)	215 (178–282)	0.402*
	Ceruloplasmin	50.29 (±9.78)	47.61 (±7.22)	0.186**
Metabolic parameters				
	Vitamin D	15.3 (4.9–36.6)	11.56 (4.5–31.3)	**0.011** *
	Insulin	15.4 (4.07–62.3)	10.8 (0.21–24.3)	**0.019** *
	HOMA-IR	3.44 (0.75–15.06)	2.37 (0.04–5.25)	**0.004** *
	HbA1c	5.2 (4.4–7.3)	5.1 (4.3–5.6)	0.073*
	Ferritin	18.4 (3.6–84.2)	11.9 (4.2–44.9)	**0.042** *
	LDL	109.5 (±37.66)	135.89 (±41.60)	**0.005** **
	HDL	66.0 (37–103)	66.0 (38–123)	0.922*
	Triglycerides	236 (120–691)	212 (65–482)	0.279*
**Perinatal and neonatal outcomes**		**GDM (n=40)**	**Control (n=39)**	
Preterm birth, n (%)		1 (2.5%)	6 (15.4%)	0.057***
Preeclampsia, n (%)		2 (5.0%)	2 (5.1%)	1.000***
FGR, n (%)		0 (0%)	1 (2.6%)	0.494***
Macrosomia, n (%)		3 (7.5%)	1 (2.6%)	0.615***
Birth weight (g), mean (±SD)		3,383.8 (±542.7)	3,301.2 (±418.8)	0.476**
Placental thickness (mm), median (min–max)		39.2 (27.6–67.5)	42.16 (25.1–68)	0.241***
NICU admission, n (%)		14 (40%)	11 (32.4%)	0.508***

Data are presented as mean±standard deviation for normally distributed continuous variables or median (minimum–maximum) for non-normally distributed continuous variables, and n (%) as appropriate. Sample size varied slightly for some laboratory and pregnancy outcomes due to missing data.

* Mann-Whitney U test.

** Independent samples t-test.

*** Fisher's exact test. GDM: gestational diabetes mellitus; OSM: oncostatin M; NLR: neutrophil-to-lymphocyte ratio; PLR: platelet-to-lymphocyte ratio; CRP: C-reactive protein; LDL: low-density lipoprotein; HDL: high-density lipoprotein; FGR: fetal growth restriction; NICU: neonatal intensive care unit; HOMA-IR: Homeostatic Model Assessment of Insulin Resistance; HbA1c: hemoglobin A1c; SD: standard deviation. Bold values indicate statistically significant p-values (p<0.05).

Perinatal and neonatal outcomes are summarized in Table 1. The incidence of preterm birth was similar between the GDM group and controls (2.5 vs. 15.4%, p=0.057). Rates of preeclampsia (5.0 vs. 5.1%, p=1.000), fetal growth restriction (FGR) (0 vs. 2.6%, p=0.494), macrosomia (7.5 vs. 2.6%, p=0.615), and neonatal intensive care unit (NICU) admission (40 vs. 32.4%, p=0.508) were comparable between the groups. Mean birth weight was similar between the GDM and control groups (3,383.8±542.7 g vs. 3,301.2±418.8 g, p=0.476). Median placental thickness was also similar between groups (39.2 vs. 42.16 mm, p=0.241) (Table 1).

Correlation analysis revealed that serum OSM levels were significantly negatively correlated with BMI (r=-0.269, p=0.019), insulin levels (r=-0.231, p=0.047), HbA1c (r=-0.376, p=0.002), and HOMA-IR (r=-0.267, p=0.021). In contrast, a significant positive correlation was observed between OSM levels and gestational age at delivery (r=0.356, p=0.002). No significant correlations were found between OSM levels and lipid parameters, including LDL cholesterol and triglycerides, nor with inflammatory markers such as CRP, ferritin, NLR, and PLR (all p>0.05) (Table 2).

**Table 2:** Correlation of serum oncostatin M levels with metabolic, inflammatory, and obstetric parameters.

	Correlation coefficient (r)	p*
BMI (kg/m^2^)	-0.269	0.019
Insulin	-0.231	0.047
HOMA-IR	-0.267	0.021
HbA1c (%)	-0.376	0.002
LDL	0.039	0.738
Triglyceride	-0.095	0.418
CRP	-0.123	0.306
Ferritin	-0.117	0.321
NLR	0.135	0.255
PLR	0.167	0.157
Gestational age at delivery (weeks)	0.356	0.002

Values are expressed as Spearman correlation coefficients.

* Statistical significance was defined as p<0.05. BMI: body mass index; LDL: low-density lipoprotein; CRP: C-reactive protein; NLR: neutrophil-to-lymphocyte ratio; PLR: platelet-to-lymphocyte ratio; HOMA-IR: Homeostatic Model Assessment of Insulin Resistance; HbA1c: hemoglobin A1c.

In a multivariable logistic regression analysis that included OSM, BMI, age, and CRP levels, none of the variables were identified as independent predictors of GDM. Lower serum OSM levels showed a trend toward association with GDM risk; however, this did not reach statistical significance (OR 0.996, 95%CI 0.991–1.001, p=0.082). The overall model demonstrated acceptable calibration (Hosmer-Lemeshow p=0.756) but limited explanatory power (Nagelkerke R^2^=0.120) (Table 3).

**Table 3:** Multivariable logistic regression analysis for predictors of gestational diabetes mellitus.

	B	SE	Odds ratio	95%CI	p
OSM (ng/L)	-0.004	0.002	0.996	0.991–1.001	0.082
BMI (kg/m^2^)	0.065	0.050	1.067	0.967–1.178	0.198
Age	-0.061	0.055	0.941	0.845–1.049	0.272
CRP	-0.003	0.054	0.997	0.896–1.108	0.950

Binary logistic regression analysis was performed to evaluate independent predictors of gestational diabetes mellitus. Odds ratios (OR) with 95%CIs were calculated. Model fit was assessed using the Hosmer-Lemeshow goodness-of-fit test. BMI: body mass index; CRP: C-reactive protein; OSM: oncostatin M; SE: standard error; CI: confidence interval.

To address the potential influence of additional metabolic covariates, separate sensitivity analyses were performed by individually adding HbA1c, fasting insulin, HOMA-IR, ferritin, vitamin D, or LDL cholesterol to the primary model. The association between OSM and GDM remained materially unchanged after adjustment for HbA1c, fasting insulin, HOMA-IR, ferritin, and vitamin D. After additional adjustment for LDL cholesterol, the association became statistically significant. The OSM odds ratios were 0.998 (95%CI 0.993–1.003; p=0.373) with HbA1c (n=59), 0.997 (95%CI 0.992–1.002; p=0.194) with fasting insulin (n=66), 0.997 (95%CI 0.992–1.002; p=0.227) with HOMA-IR (n=66), 0.996 (95%CI 0.991–1.001; p=0.085) with ferritin (n=66), 0.996 (95%CI 0.991–1.001; p=0.148) with vitamin D (n=61), and 1.005 (95%CI 1.000–1.010; p=0.048) with LDL cholesterol (n=67).

Receiver operating characteristic (ROC) analysis showed that serum OSM had a moderate discriminatory ability for GDM, with an area under the curve (AUC) of 0.707 (95%CI 0.591–0.823; p=0.001). Two OSM thresholds (30.74 and 32.09 ng/L) yielded the same maximum Youden index (0.450). We selected 32.09 ng/L for reporting because it provided higher sensitivity (57.5%) with a specificity of 87.5%. At this threshold, the positive predictive value was 82.1% and the negative predictive value was 67.3%. These findings indicate moderate discriminatory performance of OSM alone, but do not establish OSM as an independent predictor or a standalone diagnostic biomarker of GDM.

## DISCUSSION

Pregnancy represents a unique immunometabolic state characterized by dynamic immune modulation rather than uniform immune activation or suppression. Temporal shifts in inflammatory cytokine profiles throughout gestation play critical roles in maintaining maternal–fetal tolerance and supporting placental development^10–12^. OSM, a member of the interleukin-6 cytokine family, is involved in inflammatory regulation, cellular differentiation, and metabolic homeostasis, as well as placental development and endocrine regulation during pregnancy^6,13^. GDM, in contrast, is characterized by insulin resistance and metabolic dysregulation, chronic low-grade inflammation, and conditions typically associated with altered cytokine signaling^2,3,5^.

Based on these mechanisms, circulating OSM levels might theoretically be expected to increase in pregnancies complicated by GDM^5,8^. However, in the present study, maternal serum OSM concentrations were significantly lower in the GDM group compared with healthy controls. This apparently paradoxical finding may be explained by the complex immunometabolic alterations associated with insulin resistance^2,5^. Chronic metabolic stress and hyperglycemia are known to alter inflammatory and immune signaling pathways in metabolic disorders^5,14^. Therefore, decreased OSM concentrations in GDM pregnancies may reflect dysregulated cytokine signaling rather than reduced inflammation^5,8^.

Placental physiology may also contribute to these findings. The placenta represents an important source of cytokines during pregnancy, and metabolic disturbances associated with GDM may alter placental endocrine and inflammatory signaling^15^. Changes in placental cytokine production or secretion could therefore influence maternal circulating OSM concentrations. Given the expression of OSM in decidual and placental tissues, alterations in maternal circulating OSM levels may reflect changes in placental function accompanying metabolic dysfunction during pregnancy^13,15^.

The interpretation of our results is further supported by the observation that inflammatory indices, such as the NLR and PLR, were also significantly lower in the GDM group. Although previous studies have reported increased NLR and PLR in GDM, findings across populations have been inconsistent^16^. Recent studies have also highlighted associations between inflammatory and nutritional indices and adverse perinatal outcomes in diabetic pregnancies, particularly in those with pregestational diabetes^17,18^. This discrepancy may reflect differences in study populations, gestational age, and metabolic or inflammatory characteristics. GDM is generally described as a pro-inflammatory condition; however, immune responses in metabolic disorders are increasingly recognized as involving qualitative immune alterations rather than uniform quantitative increases in circulating inflammatory markers^10,12^. The concomitant reduction in leukocyte-derived inflammatory indices and OSM levels may suggest alterations in immune signaling pathways in pregnancies complicated by GDM.

Another important observation in this study was a significant negative correlation between OSM levels and metabolic parameters, including BMI, insulin, HbA1c, and HOMA-IR, and a positive correlation with gestational age at delivery. These findings suggest that OSM may be more closely associated with metabolic regulation than with systemic inflammatory activity in GDM pregnancies. Experimental studies have demonstrated that OSM plays a role in glucose homeostasis, adipose tissue metabolism, and insulin signaling pathways, supporting its potential involvement in metabolic dysfunction associated with obesity and diabetes^7,8^. The inverse relationship between OSM and metabolic severity markers observed in our cohort further supports a link between OSM and metabolic dysregulation in GDM^5,7,8,19^.

In contrast to metabolic parameters, OSM did not show significant correlations with classical systemic inflammatory markers, including CRP, ferritin, NLR, or PLR. This observation further supports the hypothesis that OSM may function at the interface between metabolism and local tissue inflammation rather than serving as a marker of systemic inflammatory burden. Such a role is consistent with emerging evidence suggesting that cytokines involved in metabolic regulation may reflect tissue-specific immune responses rather than generalized inflammation^8,9^.

Despite significant biochemical differences, perinatal outcomes were comparable between the GDM and control groups in our study. This finding is likely attributable to appropriate clinical management and glycemic control in the GDM cohort, both of which are known to reduce adverse obstetric and neonatal outcomes substantially^20^. Therefore, the absence of significant differences in neonatal outcomes does not necessarily indicate a lack of metabolic impact; rather, it reflects the effectiveness of contemporary obstetric care.

Additionally, birth weight distributions were influenced by gestational age at delivery, particularly among preterm births, which may have contributed to the absence of significant intergroup differences. Serum OSM levels were positively correlated with gestational age at delivery, suggesting that lower OSM concentrations may be associated with earlier delivery and impaired metabolic adaptation during pregnancy.

Although serum OSM levels were associated with metabolic parameters, multivariable analysis did not identify OSM as an independent predictor of GDM. This suggests that the relationship between OSM and disease status may be mediated by underlying metabolic pathways, such as adiposity and glycemic control, rather than reflecting a direct causal association^8^. Consequently, while OSM may not currently serve as a standalone predictive biomarker for GDM, it may still provide insight into the immunometabolic mechanisms underlying the disorder^21^.

Importantly, the additional sensitivity analyses requested to address potential metabolic confounding yielded consistent OSM effect estimates after adjustment for HbA1c, fasting insulin, HOMA-IR, ferritin, and vitamin D. After adjustment for LDL cholesterol, the OSM association reached statistical significance (OR 1.005, 95%CI 1.000–1.010; p=0.048). These findings should be interpreted cautiously as supportive sensitivity analyses, particularly given the complete-case sample sizes and the modest change in the OSM effect estimate.

Overall, our findings indicate that maternal serum OSM levels are reduced in pregnancies complicated by GDM and are primarily associated with metabolic status rather than systemic inflammation. These results suggest that OSM may be a component of the complex immunometabolic network underlying the pathophysiology of GDM, consistent with recent reviews emphasizing the integrated roles of metabolic, inflammatory, placental, and endocrine pathways in GDM pathogenesis^4,5,15,22^. Further prospective studies with larger cohorts are needed to clarify the mechanistic pathways and to determine whether OSM has potential diagnostic or prognostic value, particularly in relation to metabolic severity, placental function, or long-term maternal outcomes^21^.

Although maternal OSM levels were significantly lower in women with GDM, OSM was not an independent predictor in multivariable analysis and therefore cannot be considered a standalone biomarker for GDM. Future prospective studies are needed to clarify its clinical utility and potential role in multimarker risk stratification.

This study has several strengths, including being the first to evaluate maternal serum OSM concentrations in a well-characterized cohort of pregnant women with GDM and healthy controls. In addition, the simultaneous assessment of hematological inflammatory indices together with metabolic parameters provided a more comprehensive evaluation of the immunometabolic alterations associated with GDM. However, several limitations should also be acknowledged. First, this was a single-center study, which may limit the generalizability of the findings to other populations. Second, no formal a priori sample size calculation was performed, and the relatively modest sample size may have reduced the statistical power to detect associations with less frequent obstetric and neonatal outcomes. Treatment characteristics after GDM diagnosis were not systematically recorded, limiting assessment of their potential influence on OSM and metabolic parameters. Furthermore, OSM levels were measured only once during the third trimester, precluding assessment of longitudinal changes throughout pregnancy and their potential relationship with disease progression. Finally, detailed adipokine profiling and placental OSM expression were not assessed, limiting further interpretation of the mechanistic role and source of circulating OSM alterations in GDM pregnancies.

## CONCLUSION

In conclusion, maternal serum OSM levels were significantly lower in pregnancies complicated by GDM compared with healthy controls. This decrease was accompanied by reduced hematological inflammatory indices, suggesting that OSM alterations in GDM may be more closely related to immunometabolic dysregulation than to classical systemic inflammation. Despite these biochemical differences, perinatal outcomes were comparable between groups, likely reflecting effective clinical management. Our findings suggest that altered maternal serum OSM concentrations are associated with the immunometabolic changes observed in GDM. However, given the observational nature of the study, these findings should be interpreted as hypothesis-generating rather than evidence of a causal biological role. Larger prospective and mechanistic studies are needed to clarify the clinical and pathophysiological significance of OSM in GDM.

## Data Availability

The datasets generated and/or analyzed during the current study are available from the corresponding author upon reasonable request.
